# Effective Free-Driving Region Detection for Mobile Robots by Uncertainty Estimation Using RGB-D Data

**DOI:** 10.3390/s22134751

**Published:** 2022-06-23

**Authors:** Toan-Khoa Nguyen, Phuc Thanh-Thien Nguyen, Dai-Dong Nguyen, Chung-Hsien Kuo

**Affiliations:** 1 Department of Electrical Engineering, National Taiwan University of Science and Technology, Taipei 106335, Taiwan; m10907803@mail.ntust.edu.tw (T.-K.N.); d10907813@mail.ntust.edu.tw (P.T.-T.N.); d10907809@mail.ntust.edu.tw (D.-D.N.); 2 Department of Mechanical Engineering, National Taiwan University, Taipei 106319, Taiwan

**Keywords:** mobile robots, self-supervised learning, semantic segmentation, automatic labeling

## Abstract

Accurate segmentation of drivable areas and road obstacles is critical for autonomous mobile robots to navigate safely in indoor and outdoor environments. With the fast advancement of deep learning, mobile robots may now perform autonomous navigation based on what they learned in the learning phase. On the other hand, existing techniques often have low performance when confronted with complex situations since unfamiliar objects are not included in the training dataset. Additionally, the use of a large amount of labeled data is generally essential for training deep neural networks to achieve good performance, which is time-consuming and labor-intensive. Thus, this paper presents a solution to these issues by proposing a self-supervised learning method for the drivable areas and road anomaly segmentation. First, we propose the Automatic Generating Segmentation Label (AGSL) framework, which is an efficient system automatically generating segmentation labels for drivable areas and road anomalies by finding dissimilarities between the input and resynthesized image and localizing obstacles in the disparity map. Then, we train RGB-D datasets with a semantic segmentation network using self-generated ground truth labels derived from our method (AGSL labels) to get the pre-trained model. The results showed that our AGSL achieved high performance in labeling evaluation, and the pre-trained model also obtains certain confidence in real-time segmentation application on mobile robots.

## 1. Introduction

An autonomous mobile robot (AMR) is a type of robot that can understand and move through its environment independently. AMRs differ from their predecessors, autonomous guided vehicles (AGVs), which rely on tracks or pre-defined paths and often require operator oversight. Recently, AMRs have been developed for various applications that would be harmful to or not possible for human workers and reduce the labor-intensive. They may be used to clean and disinfect areas for improved health and safety, transport contagious laboratory specimens in hospitals, carry heavy loads in an industrial environment, or work in extreme conditions where humans cannot and should not be working. AMRs, nowadays, are equipped with cameras and sensors to elevate the navigation capability in their working environments and use a navigation technique such as collision avoidance to slow, stop or re-route their path around the object and then continue their task when dealing with unexpected obstacles. In addition, to prevent the AMRs from collision or turning over when tacking to obstacles, there has been a rapid development of computer vision methods in the range of research topics during recent years, such as object detection, tracking, self-localization, and lidar camera fusion, especially semantic segmentation.

Recent advancements in semantic segmentation via deep learning methods have shown fairly promising results on RGB-D datasets. A large-scale dataset with manually labeled ground truth is generally required to train a semantic segmentation network, which takes time and effort. Moreover, the development of RGB-D cameras has brought significant improvements in robotics and computer vision applications. With the ability to simultaneously transmit RGB and depth images, we use depth information to enhance the capability of segmentation resulting in detecting drivable areas and road anomalies. By taking advantage of RGB-D cameras and reducing the limitations of manual labeling, this paper proposes a self-supervised method for automatic labeling the drivable area and road obstacles for AMRs, named the Automatic Generating Segmentation Label framework (AGSL). We define the drivable area as the region through which mobile robots of any size may pass, whereas road anomalies are defined as areas taller than 5 cm above the ground.

**Figure 1:**
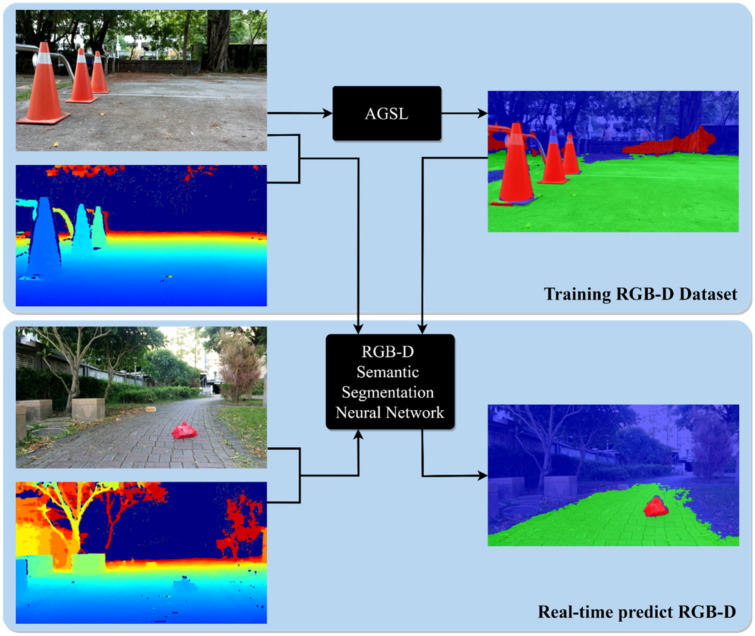
The overview of our proposed self-supervised approach for drivable area and road anomalies segmentation. We first apply AGSL framework to generate self-supervised labels (top part), then the AGSL labels are used to train the RGB-D semantic segmentation neural network. At the end, a mobile robot equipped with an RGB-D camera can perform real-time segmentation of drivable areas and road anomalies. The blue area denotes the unknown area, the green area denotes the drivable area, and the red area denotes road anomalies. The figure is best viewed in color.

Our self-supervised approach for autonomous mobile robots is inspired by [1] while extending them to utilize the benefits of uncertainty estimation [2,3,4]. The advantage of our work is that AMRs can perform the real-time segmentation for drivable area and road anomalies and also can automatically generate the self-supervised labels with the new RGB-D data inputs for future training, as shown in Figure 1. First, we develop our AGSL framework to automatically label drivable areas and road obstacles by finding the dissimilarity between the input and resynthesized image and utilizing the V-disparity map to localize road obstacles. Later, the self-generated labels and RGB-D inputs are passed through a semantic segmentation network as a training session. The pre-trained model result is used for real-time detection of drivable areas and road anomalies on mobile robots equipped with an RGB-D camera.

In addition, through extensive experiments, we demonstrate the proposed method achieves better performance compared with the baseline SSLG [1]. The pre-trained model of the FuseNet semantic segmentation neural network trained on our generated AGSL labels also exhibited effective and highly capable real-time segmentation of drivable areas and road obstacles using the RGB-D camera OAK-D in extensive testing. In summary, the contributions of this paper are following:

- This paper develops an accurate AGSL framework to automatically generate the drivable area and road anomaly maps using RGB-D data by utilizing uncertainty estimation and depth information.
- Through extensive experiments, we demonstrate that the performance of the proposed self-supervised approach containing our AGSL framework is far improved compared to an existing method. In addition, our real-time experiments showed promising feasibility in daily life environments to assist the obstacle avoidance system in improving safety.

## 2. Related Works

Image-based semantic segmentation is applicable in the scene perception of AMRs to understand the objects and obstacles in the surrounding environment to perform reliable operations. In order to exploit drivable areas and road anomalies, early approaches relied on traditional image processing algorithms. For instance, in a stereo vision-based system, e.g., [5,6,7], a disparity map and its extensions can extract a distinguishable diagonal straight line, and road obstacles can be perceived as vertical lines. However, these approaches require a relatively clean depth image as input to compute disparity with high accuracy. The other method [8] proposed a Bayesian framework to refine rich context features of scene road by using ranging (LiDAR) data and fusing it with camera information. Nevertheless, LiDAR can only work efficiently at a limited distance. Compared to expensive 3D sensors such as LiDAR, an RGB-D camera is a much lower-cost solution with higher resolution. In addition, depth maps contain more location and contour information that can be used as a critical indicator of objects in real-world scenarios. As a consequence, appropriately combining original cues (i.e., RGB) and depth is promising to improve performance.

With the rising of deep learning, many studies used a learning-based approach to segment drivable areas and road anomalies. The learning-based method transforms images into rich context features by applying advanced deep learning techniques such as data fusion-based self-supervised approach [1,9,10], uncertainty-based approach [11], and generative adversarial-based approach [4,12,13].

Recently, many awesome studies have proved that the RGB-D semantic segmentation networks with fusing depth information to the appearance can achieve better segmentation performance than single RGB-based methods. In the fusion-based self-supervised approach, recent studies have sought reliable methods to automatically label the unknown complex scenarios based on the definition of human. These methods aim to excavate the enriched perceptual features from multi-modal data fusion (e.g., RGB, Depth, LiDAR) and can work persistently under different conditions. In particular, Wang et al. [1] proposed a self-supervised approach using both RGB and depth images for the automatic labeling process. Later, RGB-D images are fed into an RGB-D semantic segmentation neural network such as FuseNet [9] to improve the performance of the drivable area and road obstacles segmentation, then achieve a pre-trained network to perform the online task. Similar to FuseNet, Sun et al. [11] designed a network that used RGB and thermal images to improve semantic segmentation in low light conditions. To this end, Lorenz et al. [10] defined drivable and obstacle detection as a classification problem. The proposed multi-model feature encoder approach is capable of projecting the input images into a feature latent space. By utilizing the training under a self-supervised approach, the distribution of safe terrain features could be desirable. Normalizing flow is utilized to transform the generated distribution and facilitate decisions, which allows tractable computation of the log-determinant of Jacobian; it requires deep networks and a large amount of training data. However, the performance of the fusion-based self-supervised approach heavily depends on the quality of generated labels, leading to some small objects and uncertain obstacles not being well determined.

In another direction, many works consider road anomaly detection as the open-set (Out-of-Distribution—OOD) semantic segmentation problem. Focusing on road regions that contains OOD obstacles, some segmentation methods tend to be overconfident to make spurious predictions. Therefore, the generated semantic map could help to reveal the unknown regions. In earlier works, Kendall et al. [14] derived the uncertainty of the segmentation map by Monte Carlo dropout down-sampling, where a higher variance of classes indicates higher uncertainty. Additionally, the uncertainty could be treated as a pixel-level uncertainty score to detect road obstacles, as introduced by [13,15].

Built on the intuition of the uncertainty in semantic segmentation, Krysztof et al. [12] detected the misclassified regions in the semantic maps as anomalies, which then leveraged the successive generative model Pix2Pix [16] model to resynthesize input from the generated semantic map. A discrepancy network is introduced to identify the difference between input and resynthesized images as the desired unknown obstacles on the road surface. However, this method performs poorly due to the uncertainty estimation being noisy and inaccurate. To overcome this limitation, methods proposed by [4,13] extracted uncertainty score maps such as softmax entropy and perceptual loss to assist the discrepancy network in generating a more meaningful anomaly map where it is easy to detect the unknown objects and road surface.

In a different approach, Lis et al. [17] proposed a method by erasing obstacles while pre-serving the road surface appearance using the GAN-based inpainting method, helping the resynthesized images illustrate more realistic in the background region. Only focusing on the drivable region, it used a network trained to find the dissimilarity between input and resynthesized road patches, making a significant increment in anomaly detection performance. Along with the GAN-based approach, the reconstructive approach can detect the road anomaly when it can reproduce the normality of the training data without any auxiliary data of anomalous objects. Vojír et al. [15] proposed a recent method that introduces the re-construction module to detect road anomalies based on pixel-wise score maps. Despite promising results, many mentioned methods in GAN-based and reconstruction-based approaches are complicated, power- and time-consuming to retrain complete models and to run on edge devices for real-world applications.

For real-world applications, to deliver a reliable but edge-device runnable model, our work savages the advantage in the performance of methods [4,13] to construct an automatic labeling system in terms of a self-supervised training pipeline. The generated labels illustrate more reliability than previous traditional methods [8,10] and are almost similar to manual labels.

## 3. Methods

**Figure 2:**
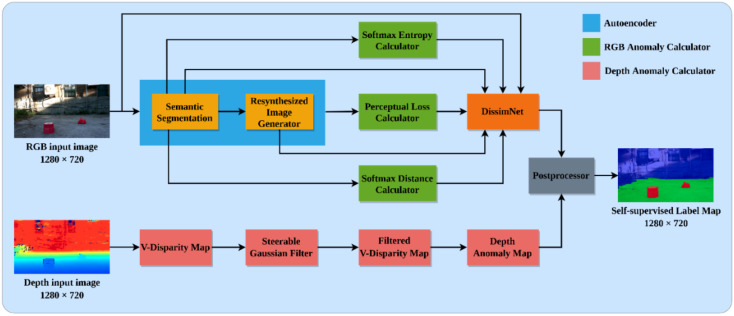
The schematic of our proposed Automatic Generating Segmentation Label system. First, we feed the RGB input image through an autoencoder, which generates a semantic map and resynthesized image. Next, an RGB anomaly calculator generates a softmax entropy, softmax distance, and perceptual loss maps from the predicted semantic map. Later, the RGB anomaly map is created by passing the produced images and the RGB input image through the spatial aware dissimilarity module (DissimNet). Additionally, we transform the depth input image to a V-disparity map and filter out noise with a steerable Gaussian filter with second derivatives. The Hough Transform algorithm is then used to extract straight lines from the filtered V-disparity map, resulting in a fast depth anomaly map. Finally, the RGB and depth anomaly maps are delivered to the post processor, which generates the self-supervised label map for drivable area and road anomalies segmentation.

In this work, we define the problem of detecting drivable areas and road anomalies using RGB-D data as the detection of ambiguous areas and road segmentation by utilizing the uncertain-ty estimation in the wrong classification of pre-trained semantic segmentation while encountering anomalous objects (i.e., a brick in the working environment) and elevate the depth information to enhance the anomalies extraction results. The proposed architecture of AGSL is illustrated in Figure 2. We briefly summarize the high-level characteristics here and discuss each part in more detail in the following subsections. The architecture can be divided into five distinct modules: auto-encoder, RGB anomaly calculator, DissimNet, depth anomaly calculator, and post-processor. In the end, the system outputs the final self-supervised label maps with drivable area and road anomaly segmentation results.

### 3.1. Autoencoder

The autoencoder comprises semantic segmentation and a resynthesized image generator. First, the autoencoder feeds the RGB input image into the semantic segmentation network by referring [18,19] to generate a semantic map. In addition, with the idea of finding the anomaly by comparing the input image and resynthesized image, we input the predicted semantic map to the resynthesized image generator to output the resynthesized image. The resynthesized image generator is trained as a conditional generative adversarial network [20,21,22]. In detail, we apply a state-of-the-art semantic segmentation neural network [23] and resynthesized network [21] trained on the Cityscapes dataset [24].

### 3.2. RGB Anomaly Calculator

Our RGB anomaly calculator combines softmax entropy, softmax distance, and perceptual loss calculators to compute diffusion measurements that quantify the uncertainty in the semantic map prediction and the essential missing information such as color or appearance between the RGB input and the resynthesized image, allowing for a direct per-pixel value comparison. First, we calculate two dispersion measures to quantify the uncertainty in the semantic map prediction. These two dispersion measurements are the softmax entropy *H* [25,26] in the softmax entropy calculator and the softmax distance *D* (i.e., the difference between the two largest softmax values) [3] in the softmax distance calculator. The softmax entropy and softmax distance of the semantic map are determined as follows for each pixel:

(1) $$H_{x}=-\underset{c\;\in \;\mathrm{classes}}{\sum }p\left(c\right)log_{2}p\left(c\right)\;$$

(2) $$D_{x}=1-\max_{c\;\in \mathrm{classes}}p\left(c\right)+\max_{c^{\prime }\;\in \mathrm{classes}\backslash (\arg\max_{c}p\left(c\right))}p\left(c^{\prime }\right)\;$$

where $p\;\left(c\right)$ is the softmax probability for class c. These two measurements are both normalized to the range of 0 and 1.

Additionally, we calculate the perceptual loss to identify the pixel with the most distinct appearance between the RGB input and the resynthesized image by using an ImageNet [27] pre-trained VGG as a feature extractor [28]. The distinction between these representations is advantageous for comparing objects on the basis of their image content and spatial organization rather than on the basis of color and texture-based low-level attributes. It is noted that if the anomaly object is not recognized or is not classified correctly, the resynthesized image is generated with the incorrect feature representation, and hence, the perceptual difference should detect these discrepancies between the RGB input image and the resynthesized image.

We define the perceptual loss between the input and resynthesized images as follows:

(3) $$V\left(I,\;R\right)=\overset{5}{\underset{i=1}{\sum }}{\left\|F^{i}\left(I\right)-F^{i}\left(R\right)\right\|}_{1}\;$$

where I and R are the RGB input and resynthesized images, respectively. Moreover, $F^{i}$ indicates the output of the *i*-th hidden layer in VGG network while $F^{i}$ (*i* = 1,…, 5) is provided by the output of the conv1_2, conv2_2, conv3_3, conv4_2, and conv5_2. The perceptual loss map is adjusted to the interval [0, 1] for consistency.

### 3.3. DissimNet

**Figure 3:**
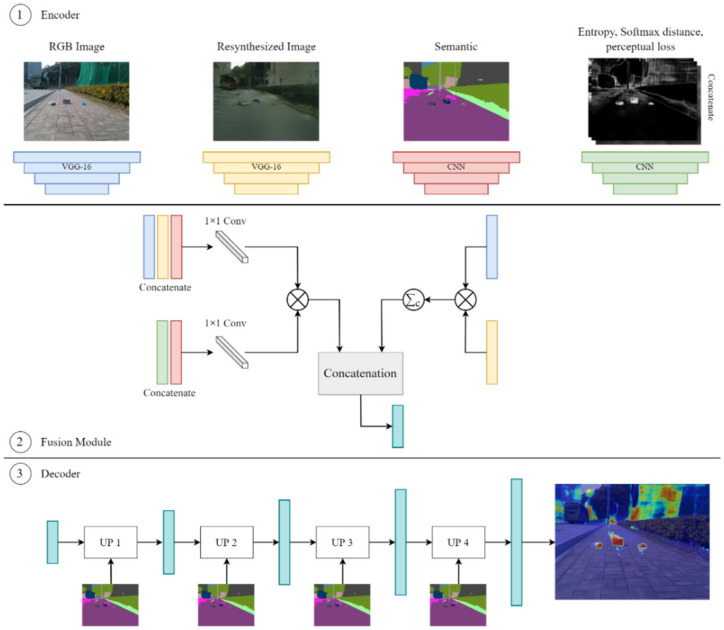
The schematic of DissimNet module aims to generate the anomaly map.

The DissimNet module takes the RGB image, semantic segmentation map, resynthesized image, as well as the ambiguous maps (softmax entropy, softmax distance, and perceptual loss) calculated in the previous step as the inputs to extract high to low-level features for each image with VGG [28] in order to generate the anomaly map. The DissimNet consists of three components (encoder, fusion module, and decoder), as seen in Figure 3.

**Encoder**. RGB image, resynthesized image, and semantic and ambiguity maps are passed through the encoder module to extract features. In this work, a pre-trained VGG-16 network is utilized to extract feature maps from RGB images and resynthesized images. In addition, first, we concatenate all uncertainty maps, including softmax entropy, softmax distance, and perceptual loss, into a three-channel uncertainty map. Later, a simple CNN network is used to encode the information from the semantic map and uncertainty image.

**Fusion Module**. In the first branch, we first concatenate the RGB input, resynthesized, and semantic feature maps and pass them through a 1 × 1 convolution. We also concatenate the uncertainty and semantic map and put it through a 1 × 1 convolution. Feature maps from two previous steps are fused at each scale by using elementwise multiplication. In the second branch, we take the elementwise multiplication between RGB and resynthesized feature maps, then followed by the channel summation. To this end, we concatenate the output feature maps of the first and second branches.

**Decoder**. We then decode each feature map of the output from the fusion module and concatenate it with the corresponding higher level in the pyramid until we get the final anomaly segmentation map. Additionally, we use the semantic map as input in the decoder module as a spatial-aware normalization presented in [29]. This normalization is utilized to ensure semantic information is not wash-away during the decoding process.

### 3.4. Depth Anomaly Calculator

Our depth anomaly calculator is inspired by [30]. As derived in [30], an RGB-D camera system consisting of two cameras, the projection of real-world point *P* with coordinates of (*X*, *Y*, *Z*) on the image coordinates (*U*, *V*) can be computed by Equations (4)–(6):

(4) $$U_{l}=u_{l}-u_{0}=f\frac{X+b/2}{Y\;\sin\;\theta +Z\cos\theta }\;$$

(5) $$U_{r}=u_{r}-u_{0}=f\frac{X-b/2}{Y\;\sin\;\theta +Z\cos\theta }\;$$

(6) $$V=v-v_{0}=f\frac{Y\;\cos\;\theta -Z\sin\theta }{Y\;\sin\;\theta +Z\cos\theta }\;$$

where b is the distance between the optical centers of two cameras; *f* is the focal length; ($u_{0},\;v_{0})$ is the center of the image plane; $u_{l},\;\;u_{r}$ are the projection of the point *P* on two cameras, respectively; θ is the pitch angle with respect to the ground plane. Then, the disparity Δ can be calculated by Equation (7):

(7) $$\Delta =u_{l}-u_{r}=f\frac{b}{Y\;\sin\;\theta +Z\cos\theta }\;$$

Labayrade et al. [30] claimed that all planes in world coordinates can be projected as straight lines in the v-disparity map. Our depth processing pipeline is based on the notion that drivable regions can be treated as planes in most circumstances and that road anomalies can be treated as planes in certain cases. The segmentation problem may therefore be approached as a straight-line extraction problem.

Depth anomaly calculation is illustrated in Figure 2. The original v-disparity map may be generated by computing the depth histogram of each row in the depth picture. However, the calculated v-disparity map typically contains a high amount of noise; the original v-disparity map is filtered using the steerable filter with Gaussian second derivatives as the basis function [7]. Straight lines may then be retrieved from the filtered v-disparity map using the Hough transform technique [31]. Gao et al. [7] discovered that the drivable region dominates the v-disparity map; the straight line with the least disparity is the projection of the infinity plane; and all other straight lines, save the two indicated above, are labeled as road anomalies. To this end, it is obvious to achieve the depth anomaly map by extracting the vertical straight lines which represent the road anomalies.

### 3.5. Post Processor

After getting the RGB anomaly map from DissimNet, road segmentation results from the semantic segmentation module, and depth anomaly map from the depth anomaly calculator, we pass them through the post-processor to process the imperfections in these maps to generate the final self-supervised label map. Let h and w denote the height and width of the input image, and all the structuring elements (i.e., a type of kernel) are square. First, we both normalize RGB and depth anomaly maps to the range [0, 1]. Then, we use the bitwise or operation to combine two anomaly maps into the final anomaly map. Later, the closing operation with structuring element size $a_{1}\times a_{1}$ is performed to the final anomaly map. Following that, the post-processor module performs a closing operation with $a_{2}\times a_{2}$ structuring element to the road segmentation map. A closing operation is a dilation followed by erosion. $a_{i}$ is generated by the following formula:

(8) $$a_{i}=f(k_{i}\times \min\left(h,\;w\right))\;\mathrm{with}\;i=1,\;2$$

where

(9) $$f\left(x\right)=2\times \left[\frac{x}{2}+1\right]-1\;$$

We find the closest odd integer to x by Equation (9). It is easier to define the origin as the center of the structuring element by assigning an odd value to $a_{i}$. A smaller $k_{i}$ results in the module detecting smaller obstacles but increasing the missing detection rate. In the controversy, a larger $k_{i}$ filters more missing detections and tiny obstacles out, but those not-so-small obstacles will also be ignored by mistake. In our experiment, the combination of $k_{1}\;$= 1/60 and $k_{2}\;$= 1/48 shows the best results.

The output final anomaly map is filtered by a pre-defined threshold to get the road anomalies and combined with the output road segmentation map as a drivable area. The area, except for road anomalies and the drivable area, is labeled as an unknown area. In the final self-supervised label map, unknown area, drivable area, and road anomalies are represented by 0, 1, and 2, respectively.

## 4. Results

In this section, we summarize the experimental results of our method on two different datasets to segment the drivable area and road obstacles without any prior knowledge about road obstacles during training.

### 4.1. GMRPD Dataset

GMRPD [1] dataset consists of 3896 RGB-D images collected by Intel Realsense D415 RGB-D camera with a resolution of 1280 × 720. It covers 30 common scenes with 18 different kinds of road obstacles that robotic wheelchairs usually cope with in a real environment. We first evaluate our method on a publicly available dataset named GMRPD, also compare our results with SSLG [1] since the GMRPD is first evaluated by this approach. In addition, we use an RGB-D data-based semantic segmentation neural networks FuseNet [9], Depth-aware CNN [32], and RTFNet [11] using ResNet18 as the backbone to evaluate our AGSL labels. After generating self-supervised labels, we train the GMRPD training set on AGSL labels. We use Stochastic Gradient Descent (SGD) with a base learning rate of 0.001 and train in 400 epochs. The resolution of the input images is downscaled to 640 × 480.

**Figure 4:**
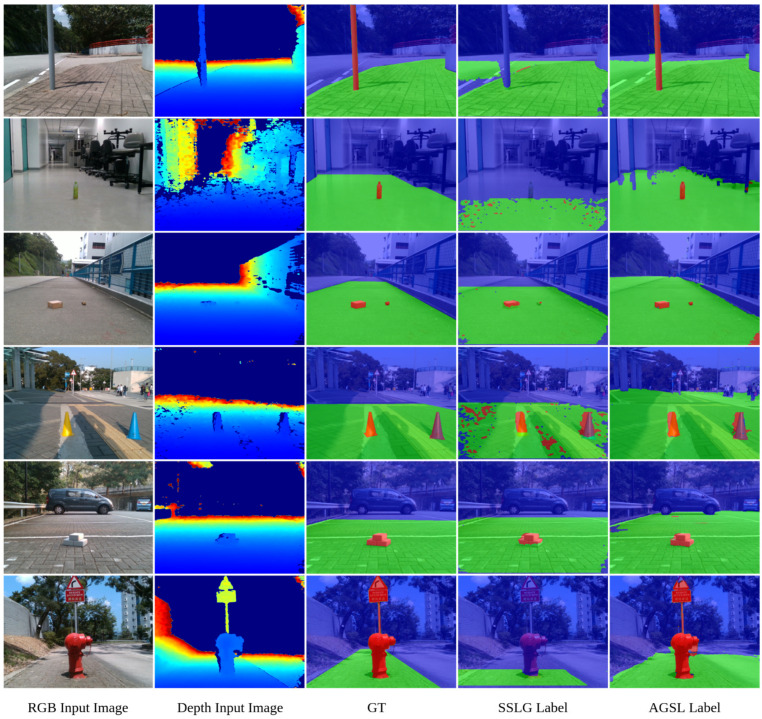
The comparison of the segmentation results between GT (manual labels), SSLG labels, and AGSL labels, respectively. The blue area denotes the unknown area, the green area denotes the drivable area, and the red area denotes road anomalies. The figure is best viewed in color.

As aforementioned, our method takes advantage of the pre-trained state-of-the-art segmentation network [23] and a resynthesized model presented by [21] trained on the Cityscapes dataset. According to experiments, the processing time to generate an AGSL label map is approximately 2 s using a GeForce RTX 2070 Graphics Card equipped with 16 GB RAM. Figure 4 shows the comparison of the segmentation results for six images. In this figure, our proposed method is indeed superior compared to the SSLG framework on the same datasets. Moreover, despite the definition of the drivable area, it can be seen from our results on GMRPD that our approach is extremely efficient in generating drivable areas and can detect road anomalies beyond the definition of manual labels. These results are also quantitatively evaluated.

The performance of the drivable area and road anomalies segmentation task is evaluated under the performance measures of precision (Pre), recall (Rec), and intersection-over-union (IoU) in three classes, including unknown area, drivable area, and road anomalies on the GMRPD dataset, as shown in Table 1. In the first part, we reimplement the SSLG method and named it SSLG++, which achieves higher results compared to the baseline with an increment of 7.13% in mean precision, 12.14% in mean recall, and 12.49% in mean IoU. It is noted that the f-number of Intel RealSense D415 is f/2.0, and the angle of view is 69°×42° (*H* × *V*). The intuition behind this improvement is that we chose the better parameters for the Hough transform in depth processing pipeline and the sigma value in the constructed Gaussian filter of the RGB processing pipeline. The limitation of the SSLG method is affected by the setting parameters for the Hough transform step and the Gaussian filter for generating the RGB anomaly map.

In addition, our AGSL with the DissimNet module is significantly better than not only the baseline (SSLG method) but also our reimplementation (SSLG++). In detail, our proposed method obtains 78.47% in mean precision, 82.99% in mean recall, and 70.03% in mean IoU, which are 12.31% (Pre), 19.59% (Rec), and 17.70% (IoU)% higher than those provided by SSLG method. The main reason behind such performance improvement is that we pay attention to high-uncertain areas in feature maps extracted from RGB input, resynthesized image, semantic map, and uncertainty maps.

In the second part, to verify the effectiveness, we show the comparison of SSLG++ labels, manual labels, and our AGSL labels on the GMRPD dataset trained on an RGB-D semantic neural network FuseNet named FSL++, FML, and FAGL; trained on Depth-aware CNN network named DSL++, DML, and DAGL; trained on RTFNet network named RSL++, RML, and RAGL, respectively. The reason we do not compare with the original SSLG labels is because of missing the detail of the validation and testing set. In this work, we use scene 12, scene 13, scene 14, and scene 29 with a total of 571 images to evaluate our training session. As shown in Table 1, the evaluation results on three off-the-shelf RGB-D networks such as FuseNet, Depth-aware CNN, and RTFNet using AGSL labels show a high increment compared to our reimplementation SSLG++, which leads to the precision improvement (Pre) of 85.03%, 86.88%, and 98.52% in terms of drivable area segmentation and 74.61%, 47.85%, and 58.32% in terms of road anomalies segmentation for FuseNet, Depth-aware CNN, and RTFNet, respectively. In addition, the results trained on AGSL labels demonstrate a comparable performance compared to those trained on manual labels.

**Figure 5:**
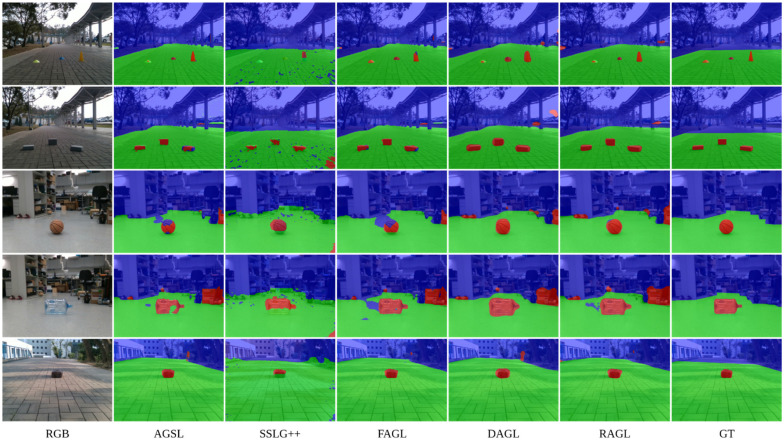
The comparison of the segmentation results between GT (manual labels), AGSL labels, SSLG++ labels, FuseNet trained on AGSL labels (FAGL), Depth-aware CNN trained on AGSL labels (DAGL), RTFNet trained on AGSL labels (RAGL), and manual labels (GT). The blue area denotes the unknown area, the green area denotes the drivable area, and the red area denotes road anomalies. The figure is best viewed in color.

In detail, FAGL reaches the precision of 74.61% in road anomalies detection compared to 82.80% of FML, and RAGL achieves a higher precision result in terms of the drivable area than RML with 98.52% vs. 97.04%. Despite our AGSL approach not yet being comparable to manual labels, it still demonstrates remarkable results using the self-supervised approach with impressive speed-up while solving the time-consuming and labor-intensive manual labeling on the GMRPD dataset, as shown in Figure 5.

### 4.2. Our Anomaly Dataset

**Figure 6:**
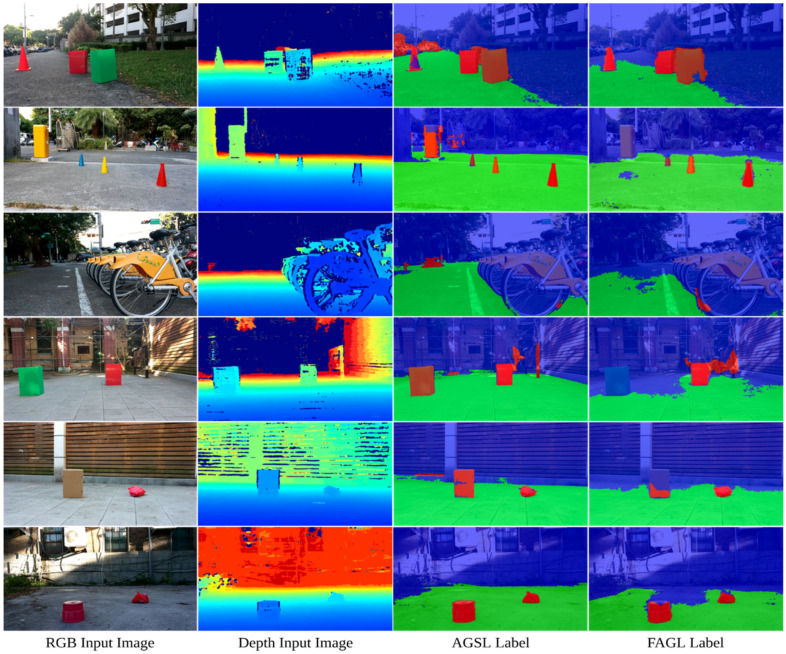
Evaluation of AGSL labels and FAGL labels (FuseNet trained on the AGSL labels) on our constructed dataset.

The proposed road anomalies segmentation method is also quantitatively evaluated as real experiments by using our anomaly dataset. Our dataset is captured under casual working conditions of mobile robots. We then use our proposed AGSL to generate the self-supervised labels with a total of 4668 images with a resolution of 1280 × 720 collected by the OAK-D camera. The dataset is split into the training, validation, and test sets containing 3268, 700, and 700 images, respectively. Each set contains different scenes from the other two sets. The FuseNet semantic segmentation network is used in the training phase and real-time segmentation evaluation. As shown in Figure 6, the results are pretty accurate compared to the human definition of road obstacles.

**Figure 7:**
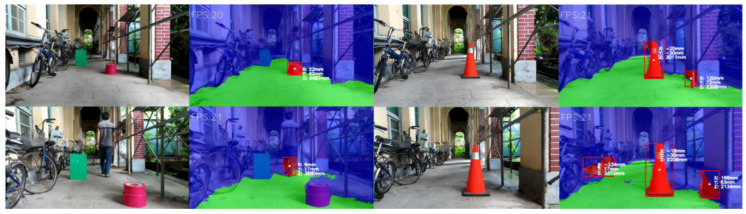
The real-time segmentation results with pre-trained FuseNet network on AGSL approach.

Furthermore, we apply the trained FuseNet network in real-time segmentation testing using a laptop equipped with GeForce GTX 1070 Max-Q, 8GB RAM while controlling our mobile robots. Our mobile robot installed an OAK-D camera as a visual perception component. The f-number of the OAK-D camera is f/2.0, and the angle of view is 69°×55° (*H* × *V*). The results show that 20 fps is the stable speed for our devices to work smoothly and perform remarkable results for segmentation and localization of road obstacles in real-world coordinates in both indoor and outdoor environments, as shown in Figure 7.

### 4.3. Limitations

Through various experimental results, the AGSL method is proved to be robust in the free-driving regions and time-saving in automatic labeling with RGB-D data as inputs. The strength of the AGSL approach is the capability to investigate the wrong prediction from the segmentation results of the state-of-the-art semantic segmentation network and find the dissimilarities between the RGB input images and resynthesize images to identify the anomalous areas. However, our proposed method is still not yet efficient enough because of the hard-code setting values in the depth anomaly calculator, such as the information of the RGB-D camera (e.g., baseline, focal length) and the angle of the Steerable Gaussian filter. Additionally, the tracing obstacles from v-disparity maps usually contain the mistaken pixels, which would lead to the increment of false negatives as low recall value in quantitative evaluation. In addition, the quality of the depth image is a critical factor that affects the AGSL system, which means it requires the RGB-D camera to be good enough to perform smoothly and effectively.

## 5. Conclusions

This study proposes a comprehensive solution to identify the segmentation of drivable areas and road anomalies (obstacles) for autonomous mobile robots. A self-supervised technique was proposed consisting of an automatic labeling pipeline to segment drivable areas and road obstacles. AGSL system composes of an RGB anomaly calculator and depth anomaly calculator, which is a practical solution to reduce time and labor-intensive manual labeling. Especially, our approach utilizes the uncertainty maps (i.e., softmax entropy, softmax distance, and perceptual loss maps) to localize the road anomalies based on the difference between the input and resynthesized images in terms of unknown objects. Our experimental results demonstrated that the proposed method significantly speeds up compared to manual labeling. In addition, our AGSL method is significantly improved compared with the SSLG method on the GMRPD benchmark. In future works, we consider designing a framework totally based on deep learning techniques using RGB-D data to reduce the limitation of extracting information from depth images based on traditional image processing and investigate the path planning algorithm based on segmentation. Last but not least, we demonstrated robust and accurate real-time segmentation applications on a mobile, which is the potential to combine with path planning to improve the automatic navigation of autonomous mobile robots.

## Figures and Tables

**Table 1:** The comparison of the segmentation results (%) between SSLG labels, SSLG++ (our reimplementation), AGSL labels, FuseNet trained on SSLG++ labels (FSL++), FuseNet trained on AGSL labels (FAGL), FuseNet trained on manual label (FML), Depth-aware CNN trained on SSLG++ labels (DSL++), Depth-aware CNN trained on AGSL labels (DAGL), Depth-aware CNN trained on manual label (DML), RTFNet trained on SSLG++ labels (RSL++), RTFNet trained on AGSL labels (RAGL), and RTFNet trained on manual label (RML). Best results without using manual labels are highlighted in bold font.

Approach	Unknown Area			Drivable Area			Road Anomalies			All		
	Pre	Rec	IoU	Pre	Rec	IoU	Pre	Rec	IoU	Pre	Rec	IoU
SSLG	89.62	80.36	75.09	75.70	86.91	65.87	33.15	22.92	16.03	66.16	63.40	52.33
SSLG++	**97.82**	90.25	**87.80**	88.63	**96.46**	**84.42**	33.41	39.91	22.23	73.29	75.54	64.82
AGSL	94.63	**90.30**	85.90	**88.75**	93.22	83.37	**52.04**	**65.46**	**40.83**	**78.47**	**82.99**	**70.03**
FSL++	99.67	87.58	87.32	83.59	**97.99**	82.17	29.26	43.90	21.30	70.84	76.49	63.60
FAGL	95.65	89.30	85.82	85.03	93.69	80.42	**74.61**	72.44	**58.12**	**85.10**	85.14	74.79
FML	98.59	99.13	97.75	98.69	97.37	96.13	82.80	99.63	82.55	93.36	98.71	92.14
DSL++	**99.83**	85.77	85.64	81.49	97.13	79.58	26.72	50.73	21.21	69.35	77.88	62.14
DAGL	98.71	90.11	89.07	86.88	96.34	84.10	47.85	**93.18**	46.23	77.81	93.21	73.13
DML	98.44	96.96	95.50	95.40	97.03	92.69	78.11	96.23	75.79	90.65	96.74	87.99
RSL++	99.37	98.07	**97.47**	97.64	97.51	**95.26**	54.04	84.78	49.26	83.68	**93.45**	**80.66**
RAGL	95.61	**98.87**	94.58	**98.52**	94.03	92.72	58.32	76.44	49.44	84.15	89.78	78.91
RML	99.58	98.00	97.60	97.04	98.69	95.80	77.52	99.64	77.30	91.38	98.78	90.23

## Data Availability

Not applicable.
